# Annotations

**Published:** 1900-08-11

**Authors:** 


### Annotations.

### Sunstroke.

Much work has been done lately in the direction of elucidating the pathology of sunstroke, but it cannot be said that as yet any very firm or satisfactory conclusion has been arrived at. It has long been held that insolation, sunstoke, heat stroke, thermic fever, or siriasis, as some prefer to call it, is due to the exposure of the body to excessive heat, but even if that be granted, investigators are by no means unanimous as to the manner in which the symptoms are produced, whether, that is, they are the immediate consequences of the action of undue heat upon the tissues, notably the nervous structures, or of changes in the blood caused in the same way; or are the result of a poisoning of the system by some toxic product, an acute toxæmia arising suddenly in the body under the influence of heat, and acting with destructive energy upon the cells of the nervous system, especially those governing the heart and controlling the vaso-motor apparatus, or finally whether they arise from an infection which only operates in the presence of high temperature. We, at any rate, seem to have got so far as this, that the blood of patients suffering from insolation does seem to contain a toxic substance which can be shown by experiments on animals to be somewhat analogous in its action to snake poison. But whether that is caused directly by heat, or is the result of some ferment, and, if the latter, whether this ferment arises from within or is an infection from without, are questions as yet quite undecided.

### The Doctor and the Nurse.

Nurses are indispensable. They are helpful, they cheer the patient, they lighten the labour of the doctor, and are in every way to be desired. Nevertheless, even the sweetest of God's gifts have their little drawbacks, and it is to be feared that constant dependence on the nurse is somewhat enervating to the doctor. Theoretically, the nurse's duty is to carry out the directions of the medical practitioner, so that his treatment, which is the outcome of his vast knowledge and experience, shall not lose in efficacy by being clumsily applied by the "untrained" hands of mothers, or wives, or other such ignorant people. Practically, it is to be feared that nurses are too often just a simple way of saving trouble. Not that we grumble or, indeed, dislike the process. It is very nice to have the temperature taken for one, to learn by a glance at the card all sorts of monotonous details, to find the dressings ready, to have the water poured into the basin, and the towel handed to one; and then it certainly is a great facility and an unspeakable saving of time and worry to be able to dismiss in a few words all such matters as a nurse is imagined to know about. "Be careful as to food, you know." "If she shivers you know what to do," and so on, are things easy to say, and if the nurse is really up to her work are no doubt sufficient. But to treat one's patient in this way is vastly enervating to the doctor, and not altogether free from danger to the patient. Certain details of management, or, as we should prefer to call it, of treatment, have come by common consent to be relegated almost entirely to the nurse, and her department is, we fancy, becoming wider every year; all of which tends to lax and flabby treatment. In certain special hospitals, where the same men are working constantly with the same nurses, it would no doubt be ridiculous to repeat the same instructions in case after case. But when a practitioner takes his chance of a nurse, trained he knows not where, or according to what rules, he will, if he is wise, keep a firm hand, a firmer hand than some nurses altogether like, on what may by some be regarded as mere nursing details. Doctors sometimes grumble at the presumption of nurses, but in truth it is often their own fault. The medical practitioner is responsible for the whole treatment of his patient, and the nurse's place ought to be to carry out the details of the treatment according to his instructions. Too often, it is to be feared, he makes the presence of the nurse an excuse for not giving these instructions, and for not thinking out these details. When the diet, the bowels, the back, the sponging, the clothing, the temperature and ventilation, the number of visitors, the length of their stay, the sitting up, the lying down, and all such things—even the night draught and the morning dose—are left to the nurse, we need hardly be surprised that sometimes the friends are apt to wonder whether the daily visit of the doctor who merely glances at the card and says, "Go on," is altogether worth the fee. Now, the doctor of the old school, before trained nurses occupied their present position, inquired after or gave directions about each of these things at every visit, and was a much more essential person.

### Ladies as Infirmary Visitors.

It is difficult to understand what can have induced the governors of the Wigan Infirmary to take the action which they did last week in refusing to allow the appointment of ladies as visitors of the infirmary. It seems that an attempt was made at the last annual meeting to place two ladies on the list of visitors, when it was found that the rules did not provide for any such innovation. It was, therefore, determined by those in sympathy with the proposal to effect the necessary alteration in the rules, and for this purpose a special meeting was called together on their requisition. It is evident that the question has excited some interest in the town, and we understand that a somewhat lively discussion was anticipated. The Mayor, however, suggested that as soon as the mover and seconder of the proposal had spoken the vote should be taken, and when this was done it resulted in an overwhelming defeat for the requisitioners and their friends. When we find women taking the active part they now do in public affairs, and when we see the good work they are doing on boards of guardians, on the Metropolitan Asylums Board, as inspectors of factories, as sanitary inspectors, and in many other posts, it is sufficiently surprising to find that the rules of many hospitals do not admit of their occupying seats upon their boards of management. But to refuse to accept them as visitors even is certainly a far worse step, and is a strange commentary upon the position of women in the North of England.

